# Silencing of the Host Factor *eIF(iso)4E* Gene Confers *Plum Pox Virus* Resistance in Plum

**DOI:** 10.1371/journal.pone.0050627

**Published:** 2013-01-28

**Authors:** Xinhua Wang, Susanne E. Kohalmi, Antonet Svircev, Aiming Wang, Hélène Sanfaçon, Lining Tian

**Affiliations:** 1 Department of Biology, University of Western Ontario, London, Ontario, Canada; 2 Southern Crop Protection and Food Research Centre, Agriculture and Agri-Food Canada, London, Ontario, Canada; 3 Pacific Agri-Food Research Centre, Agriculture and Agri-Food Canada, Summerland, British Columbia, Canada; National Taiwan University, Taiwan

## Abstract

*Plum pox virus* (PPV) causes the most economically-devastating viral disease in *Prunus* species. Unfortunately, few natural resistance genes are available for the control of PPV. Recessive resistance to some potyviruses is associated with mutations of eukaryotic translation initiation factor 4E (eIF4E) or its isoform eIF(iso)4E. In this study, we used an RNA silencing approach to manipulate the expression of *eIF4E* and *eIF(iso)4E* towards the development of PPV resistance in *Prunus* species. The *eIF4E* and *eIF(iso)4E* genes were cloned from plum (*Prunus domestica* L.). The sequence identity between plum *eIF4E* and *eIF(iso)4E* coding sequences is 60.4% at the nucleotide level and 52.1% at the amino acid level. Quantitative real-time RT-PCR analysis showed that these two genes have a similar expression pattern in different tissues. Transgenes allowing the production of hairpin RNAs of plum *eIF4E* or *eIF(iso)4E* were introduced into plum via *Agrobacterium*-mediated transformation. Gene expression analysis confirmed specific reduced expression of *eIF4E* or *eIF(iso)4E* in the transgenic lines and this was associated with the accumulation of siRNAs. Transgenic plants were challenged with PPV-D strain and resistance was evaluated by measuring the concentration of viral RNA. Eighty-two percent of the *eIF(iso)4E* silenced transgenic plants were resistant to PPV, while *eIF4E* silenced transgenic plants did not show PPV resistance. Physical interaction between PPV-VPg and plum eIF(iso)4E was confirmed. In contrast, no PPV-VPg/eIF4E interaction was observed. These results indicate that eIF(iso)4E is involved in PPV infection in plum, and that silencing of *eIF(iso)4E* expression can lead to PPV resistance in *Prunus* species.

## Introduction

*Plum pox virus* (PPV) causes disease in *Prunus* trees, including peaches, plums, apricots, cherries and ornamental species. Plum pox, also known as Sharka, is the most devastating *Prunus* viral disease in terms of economic and agronomic importance worldwide [1]–[3]. The disease was first reported in Bulgaria in 1917, although its viral nature was not identified until 1932 [4]. It causes fruit acidity and deformation, rendering the fruit unsuitable for direct consumption and processing, and premature fruit drop [5]. PPV can cause devastating yield loss of fruit crops of up to 100% [5].

PPV belongs to the genus *Potyvirus*, family *Potyviridae*. The PPV genome consists of a positive-sense single-stranded RNA molecule approximately 9.8 kb in length and has a 5′-terminal VPg (viral protein genome-linked and a 3′- polyadenylated tail [6], [7]. To date, seven PPV strains have been found: PPV-D (Dideron), PPV-M (Marcus), PPV-C (Cherry), PPV-EA (EI Amar) [2], PPV-Rec (recombinant) [8], PPV-W [9] and PPV-T (Turkey) [10]. PPV is transmitted by aphids over short distances in a non-persistent manner. Long distance transmission occurs mainly through distribution and propagation of infected nursery stocks to new locations [1], [11].

Full resistance to PPV has not yet been achieved in *Prunus* species in spite of many years of extensive breeding programs. To date, there is no effective method to cure or treat PPV infected trees. PPV has spread to most European countries and in recent years has been found in many other countries, including India [12], China [13], Japan [14], the United States [15] and Canada [16].

The lack of natural resistant germplasms makes genetic engineering an important alternative approach to develop PPV resistance in plants. Transgenes expressing different segments of the PPV genome have been used to induce PPV-specific RNA silencing and to confer resistance to PPV in model plants and in plum [17]–[22]. Stable transgenic PPV resistance was also observed in field trials [23], [24]. Thus, PPV resistance can be successfully achieved via transgenic technology in its natural woody host. Honeysweet PPV resistant genotype, generated via above approach, has been intensively tested and evaluated for the general biology characteristics and the regulation papers regarding field growth of the plants for PPV resistance have also been processed (R. Scorza, person communication). No doubt, this is an effective method for generating PPV resistant plants. Nevertheless, certain limitation exists in viral-derived resistance. Introduction of PPV genome segments into plants might be viewed with concern by the public. It has been suggested that recombination of the introduced viral genome segments with the genome of other infecting viruses, could lead to the development of new viruses [25]–[28]. Also, virus-based resistance is often narrow and plants could still be susceptible to divergent viral strains.

Viruses encode a limited number of proteins and depend on the recruitment of host factors to complete their life cycle. These host factors are potential targets for alternative antiviral strategies. Many antiviral recessive resistance genes encode components of the translation initiation complex, including the eukaryotic translation initiation factor 4E (eIF4E) and eIF4G and their isoforms [29]–[35]. eIF4E is a cap-binding protein that interacts with the 5′ cap structure of mRNAs and mediates recruitment of mRNAs to the ribosome [36]. eIF4E is associated with eIF4G, a scaffold protein, and eIF4A, an RNA-dependent ATPase and RNA helicase, to form the eIF4F complex [37]–[39]. A direct interaction between eIF(iso)4E and a potyvirus VPg protein was identified [40]. The interaction between eIF(iso)4E and VPg correlates with potyvirus infectivity and the abolishment of this interaction can lead to virus resistance [30], [41]. The involvement of eIF4E and/or eIF(iso)4E in potyvirus infections has been reported in several plant species, including *Arabidopsis thaliana*, lettuce, pepper and pea [29], [31], [32], [42]. eIF4E and/or eIF(iso)4E were also identified as susceptibility factors required for infection of cucumoviruses [43], bymoviruses [44] and carmoviruses [45]. The requirement for either eIF4E or eIF(iso)4E varies with the plant and virus considered. An *Arabidopsis* mutant lacking eIF(iso)4E showed resistance to PPV [46], suggesting that eIF(iso)4E may play an important role for PPV infection. In apricot, a quantitative trait loci associated with PPV resistance was found to colocalize with eIF4E, although the role of this co-factor in virus resistance is not known [47].

In this study, the involvement of plum eIF4E and eIF(iso)4E in PPV infection was investigated in its natural host plum (*P. domestica*) by specifically silencing either *eIF4E* or *eIF(iso)4E*. The results indicated that silencing of *eIF(iso)4E* but not *eIF4E* provided effective resistance to PPV in *Prunus* species. This is the first report confirming the involvement of eIF(iso)4E in PPV infection in its natural woody host.

## Results

### Cloning and sequencing of plum *eIF4E* and *eIF(iso)4E* genes

To design primers for cloning of *eIF4E* and *eIF(iso)4E* genes from plum, *eIF4E* sequences for peach (AJ823667), apple (CV149907), apricot (CO370600) and *Arabidopsis* (Y10548) and *eIF(iso)4E* sequences for peach (DY638147), lettuce (AAP86603) and *Arabidopsis* (Y10547) were aligned and compared (data not shown). Conserved sequences were chosen for primer pairs Pd4E-F1 and Pd4E-R1, and Pdiso-F5 and Pdiso-R1 (Table 1) to amplify plum *eIF4E* and *eIF(iso)4E*, respectively. Reverse transcription (RT)-PCR was conducted using total RNA isolated from plum leaves. Amplified PCR products of the expected length were cloned and sequenced. The sequences were used to design new primers (Table 1) to obtain the full length cDNA sequences using 5′-RACE and 3′-RACE. The sequence of the putative *eIF4E* gene was verified by amplifying a nearly full length cDNA using primers specific for the 5′- untranslated region (UTR, primer Pd4E-F2) and 3′-UTR (Pd4E-R3) (Table 1). The assembled *eIF4E* cDNA sequence was 960 bp long, including a coding region (CDS) of 702 bp, a 5′-UTR of 85 bp and a 3′-UTR of 173 bp. The predicted protein has 233 amino acid residues with a calculated molecular mass of 26.4 kDa. The sequence similarity of the cloned gene to other *eIF4Es* was confirmed using BLAST. The closest matches with the predicted translation product were eIF4E from pea (*Pisum sativum*; AAR04332; E = 8e^−92^), soybean (*Glycine max*; ACM45958; E = 5e^−90^) and muskmelon (*Cucumis melo*; ABD57970; E = 1e^−86^).

**Table 1:** Sequences of primers used in this study.

Primer name	Primer sequence (5′- 3′)
Pd4E-F1	TAGCCCCGCAGCAAAGTCC
Pd4E-R1	CTTCCACTGTTTCCCAATGCTCA
Pdiso4E-F5	TGGTTCGATAACCAATCCAAGC
Pdiso4E-R1	CTCATCAGCCTCATCAAATTGCTC
Pd4E-5′-RACE-Outer primer	ACAGCCAAGAGGTATCAGATTTCC
Pd4E-5′-RACE-Inner primer	CCTTTAGGTAAAGTTACAGTCCAC
Pd4E-3′-RACE-Outer primer	GATCATGGAGATGAAATTTGTGGAG
Pd4E-3′-RACE-Inner primer	TGGAGCAGTTGTCAACGTCAGA
Pdiso4E-5′-RACE-Outer primer	AAGCAGCACCTTGCTTGGGCTT
Pdiso4E -5′-RACE-Inner primer	TGCTTGGGCTTGGATTGGTTATC
Pdiso4E -3′-RACE-Outer primer	CAAGCCGAGCAAGTTTCCACCA
Pdiso4E -3′-RACE-Inner primer	CAAATGCAGATTTCCACTTGTTCAG
Pd4E-Fm3	GGAGCAGTTGTCAACGTCAG
Pd4E-Rm3	GTAATCCAGAAGCCCCTTCC
Pdiso-Fm3	AGGCAGGACAAACTTGCATT
Pdiso-Rm3	CGAGGCTTTGCTGATCTTTC
Ps-actin-F	CTGGACCTTGCTGGTCGT
Ps-actin-R	ATTTCCCGCTCAGCAGTG
Pd4E-attB1	GGGGACAAGTTTGTACAAAAAAGCAGGCTTCATGG TGGTCGAAGACGCACT
Pd4E-attB2	GGGGACCACTTTGTACAAGAAAGCTGGGTCGTAAA TGCTCCAGAACTCCTCG
Pdiso-attB1	GGGGACAAGTTTGTACAAAAAAGCAGGCTTCA TGGCGACAGAGGTAGCAG
PdisoattB2	GGGGACCACTTTGTACAAGAAAGCTGGGTCTGG TGGAAACTTGCTCGGCT
nptII-F	GAGGCTATTCGGCTATGACTG
nptII-R	ATCGGGAGCGGCGATACCGTA
35S-F	CTTCGCAAGACCTTCCTCT
Pd4E-siR	GTAAATGCTCCAGAACTCCTCG
Pdiso-siR	TGGTGGAAACTTGCTCGGCT
Pd4E-F8	ATGGTGGTCGAAGACGCACT
Pd4E-R2	GAACAATATACACATCAGGCTACG
Pd4E-F2	CACAAAACGAAACGCCAAGAAAG
Pd4E-R3	TTGTAGAAAGTAAACAGCTCATATCC
Pdiso-F1	ATGGCGACAGAGGTAGCAG
Pdiso-F2	ACCCCGAGAGACATACAGAC
Pdiso-R2	GTGACGGTGTTCACAACTTTGG
Pdiso-R3	TTACAAACTAAACATACTTTTTAAGTATAC
Pd4E-attB2-Y2H	GGGGACCACTTTGTACAAGAAAGCTGGGTCGACTACGTA TTTATTTTTGGC
Pdiso4E-attB2-Y2H	GGGGACCACTTTGTACAAGAAAGCTGGGTCAACATTGTA TCGAGGCTTTGC
VPg-attB1	GGGGACAAGTTTGTACAAAAAAGCAGGCTTCATGGGCTT CAATCGTAGGCAAAGA
VPg-attB2	GGGGACCACTTTGTACAAGAAAGCTGGGTTCTCGTGGTCAACTTCTTCGTC

OP: Outer Primer, IP: Inner Primers, Underline: *att*B recombination sites.

Similarly, the cDNA sequence of plum *eIF(iso)4E* was obtained by assembling the sequences of 5′-RACE and 3′-RACE and verified by RT-PCR using primers of Pdiso-F2 and Pdiso-R3 (Table 1). The cDNA sequence obtained is 990 bp in length, including a CDS of 642 bp, a 5′-UTR of 88 bp, and a 3′-UTR of 260 bp. The gene is predicted to encode a 213 amino acid protein with a calculated molecular mass of 24.1 kDa. In BLAST searches, the closest matches to the predicted eIF(iso)4E protein were eIF(iso)4E protein from grape (*Vitis vinifera*, XP_002285444; e = 1e^−94^), eIF4E protein from castor bean (*Ricinus communis*; XP_002528368; E = 5e^−89^) and eIF(iso)4Es from lettuce (*Lactuca sativa*; AAP86603; E = 8e^−88^) and cucumber (*Cucumis sativus*; ABY56102; E = 8e^−88^), suggesting that the cloned gene is *eIF(iso)4E*. The cloned plum *eIF4E* and *eIF(iso)4E* genes were designated *PdeIF4E* and *PdeIF(iso)4E*. The sequence identity between *PdeIF4E* and *PdeIF(iso)4E* coding sequences is 60.4% at the nucleotide level and 52.1% at the amino acid level. Both *PdeIF4E* and *PdeIF(iso)4E* sequence data have been deposited into GeneBank (accession numbers are JX137116 for *PdeIF4E* and JX137117 for *PdeIF(iso)4E*).

**Figure 1:**
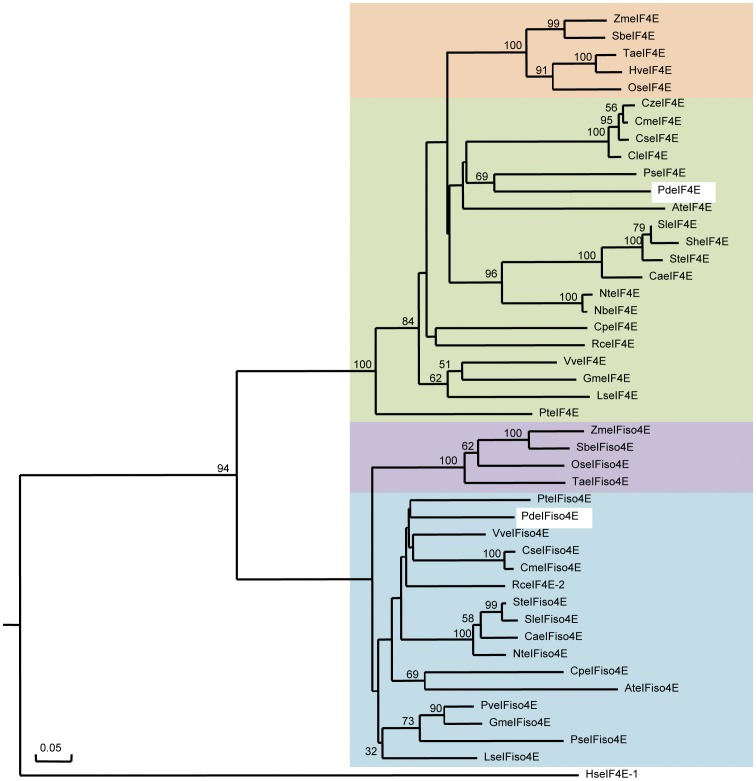
Phylogenetic analysis of eIF4E and eIF(iso)4E from different plant species. The rooted phylogenetic tree was generated with DNAman using a bootstrap value of 1000. The tree uses full length amino acid sequences of eIF4E and eIF(iso)4E from different plant species. Numbers at the branch points identify the boot strap values. The human HseIF4E-1 was used as an outlier. The name and accession number of amino acid sequences used for phylogenetic analysis are: *Prunus domestica* (PdeIF4E, JX137116; PdeIF(iso)4E, JX137117), *Pisum sativum* (PseIF4E, AAR04332; PseIF(iso)4E, ABH09880), *Glycine max* (GmeIF4E, ACM45958; GmeIF(iso)4E, ACU23400), *Vitis vinifera* (VveIF4E, XP_002267488; VveIF(iso)4E, XP_002285444), *Cucumis melo* (CmeIF4E, ABD57970; CmeIF(iso)4E ABY56090), *Populus trichocarpa* (PteIF4E, XP_002316746; PteIF(iso)4E, XP_002312598), *Citrullus lanatus* (CleIF4E, ACN51299), *Cucumis zeyherii* (CzeIF4E, ABS18380), *Cucumis sativus* (CseIF4E, ABY56085; CseIF(iso)4E, ABY56102), *Ricinus communis* (RceIF4E, XP_002519771; RceIF4E-2, XP_002528368), *Carica papaya* (CpeIF4E, ACN38307; CpeIF(iso)4E, ACM18197), *Lactuca sativa* (LseIF4E, AAP86602; LseIF(iso)4E, AAP86603), *Nicotiana tabacum* (NteIF4E, CBJ34332; NteIF(iso)4E, AAU06579), *Arabidopsis thaliana* (AteIF4E, NP_193538; AteIF(iso)4E, NP_198412), *Nicotiana benthamiana* (NteIF4E, ABD57972), *Zea mays* (ZmeIF4E, ABD57972; ZmeIFiso4E, ACG47262), *Sorghum bicolour* (SbeIF4E, XP_002457018; SbeIF(iso)4E, XP_002467110), *Solanum tuberosum* (SteIF4E, CBJ34334; SteIF(iso)4E, CBJ34336), *Capsicum annuum* (CaeIF4E, AAN74644; CaeIF(iso)4E, AAY62607), *Oryza sativa* (OseIF4E, NP_001045525; OseIFiso4E, NP_001064810), *Solanum habrochaites* (*SheIF4E*, AAV88613), *Solanum lycopersicum* (SleIF4E, ABF83563; SleIF(iso)4E, ABV23495), *Triticum aestivum* (TaeIF4E, P29557; TaeIF(iso)4E, Q03389), *Hordeum vulgare* (HveIF4E, AAV80393), *Phaseolus vulgaris* (PveIF(iso)4E, ABU54805). To distinguish between the two RceIF4Es, XP_002528368 was labelled as RceIF4E-2 in the alignment. eIF4E and eIF(iso)4E sequences from different plant groups are color-coded (green: dicot eIF4E sequences; orange: monocot eIF4E sequences; blue: dicot eIF(iso)4E sequences; purple: monocot eIF(iso)4E sequences). The two plum sequences are highlighted in white.

To compare the relationships of eIF4E superfamily members from different plant species, full length protein sequences of eIF4E and eIF(iso)4E were aligned and a phylogenetic analysis was conducted. Two distinct branches were formed in the phylogenetic tree (Figure 1) with all eIF4E sequences, except for ReeIF4E-2, in one branch and all eIF(iso)4E sequences in a second branch. The alignments suggest that ReeIF4E-2 represents an eIF(iso)4E instead of an eIF4E as annotated in GeneBank. In the eIF4E branch, two obvious subgroups can be identified representing sequence from monocots or dicots. As expected, PdeIF4E grouped together with the dicots species sequences (Figure 1). Similar results were obtained for eIF(iso)4E, as sequences from monocots and dicots formed distinct subgroups (Figure 1).

### Gene expression profile of *PdeIF4E* and *PdeIF(iso)4E* in different tissues

Gene specific primer pairs Pd4E-Fm3/Pd4E-Rm3, and Pdiso4E-Fm3/Pdiso-Rm3 (Table 1) were used to amplify pPdeIF4E and pPdeIF(iso)4E, respectively. Gel electrophoresis confirmed that primer pair Pd4E-Fm3/Pd4E-Rm3 amplified a fragment of the expected size (117 bp) from pPdeIF4E but not from pPdeIFiso4E. Similarly, Pdiso4E-Fm3/PdisoRm3 amplified a fragment of the expected size (154 bp) from pPdeIF(iso)4E but not from pPdeIF4E (not shown).

**Figure 2:**
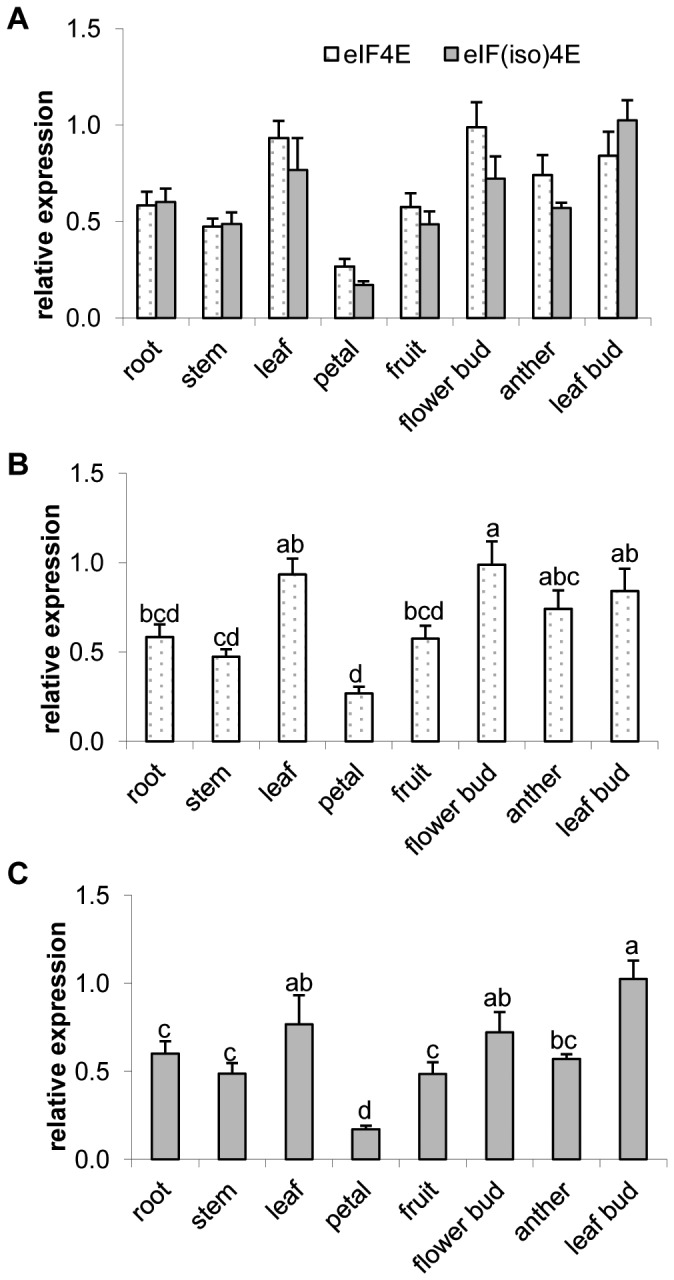
mRNA transcript levels for *PdeIF4E* and *PdeIF(iso)4E* in different tissues. Relative expression levels were determined by real-time PCR using a standard curve approach. The values represent means of three biological repeats and the value of each biological repeat is the mean of three technical repeats. All values were normalized to the reference gene *Ps-actin*. The raw numerical data were analyzed by ANOVA and the means compared with Duncan's Multiple Range Test with SAS software (SAS 9.1). Letters a, b, c and d indicate the statistic difference between samples at *P* = 0.05. Means with the same letter are not significantly different. A. *PdeIF4E* and *PdeIF(iso)4E* mRNA expressions in different tissues. B. Statistical comparison of *PdeIF4E* mRNA expression in different tissues. C. Statistical comparison of *PdeIF(iso)4E* mRNA expression in different tissues.

Expression profiles for *PdeIF4E* and *PdeIF(iso)4E* were examined in different plum tissues using quantitative real-time PCR. Total RNA was isolated from roots, stems, leaves, petals, green immature fruit, flower buds, anthers and leaf buds of wild type plum trees. Expression levels of *PdeIF4E* and *PdeIF(iso)4E* were normalized to the expression level of an internal control gene. The *Ps-actin* gene was chosen as a control as its expression is consistent under different conditions in different tissues [48]. The expression patterns of *PdeIF4E* and *PdeIF(iso)4E* were very similar in different tissues (Figure 2A). When comparing the mRNA transcript levels of both genes in the same tissue, no significant differences were detected. However, mRNA transcript levels were significantly different in different tissues when comparing data for a single gene (Figure 2B, 2C). The highest level of *PdeIF4E* mRNA was found in flower buds, followed by leaves, leaf buds and anthers (Figure 2B). Similarly, highest levels of *PdeIF(iso)4E* mRNA were found in leaf buds, leaves and flower buds (Figure 2C). Lowest expression was in petals for both genes (Figure 2).

### Genetic transformation and initial characterization of transgenic plum plants

**Figure 3:**
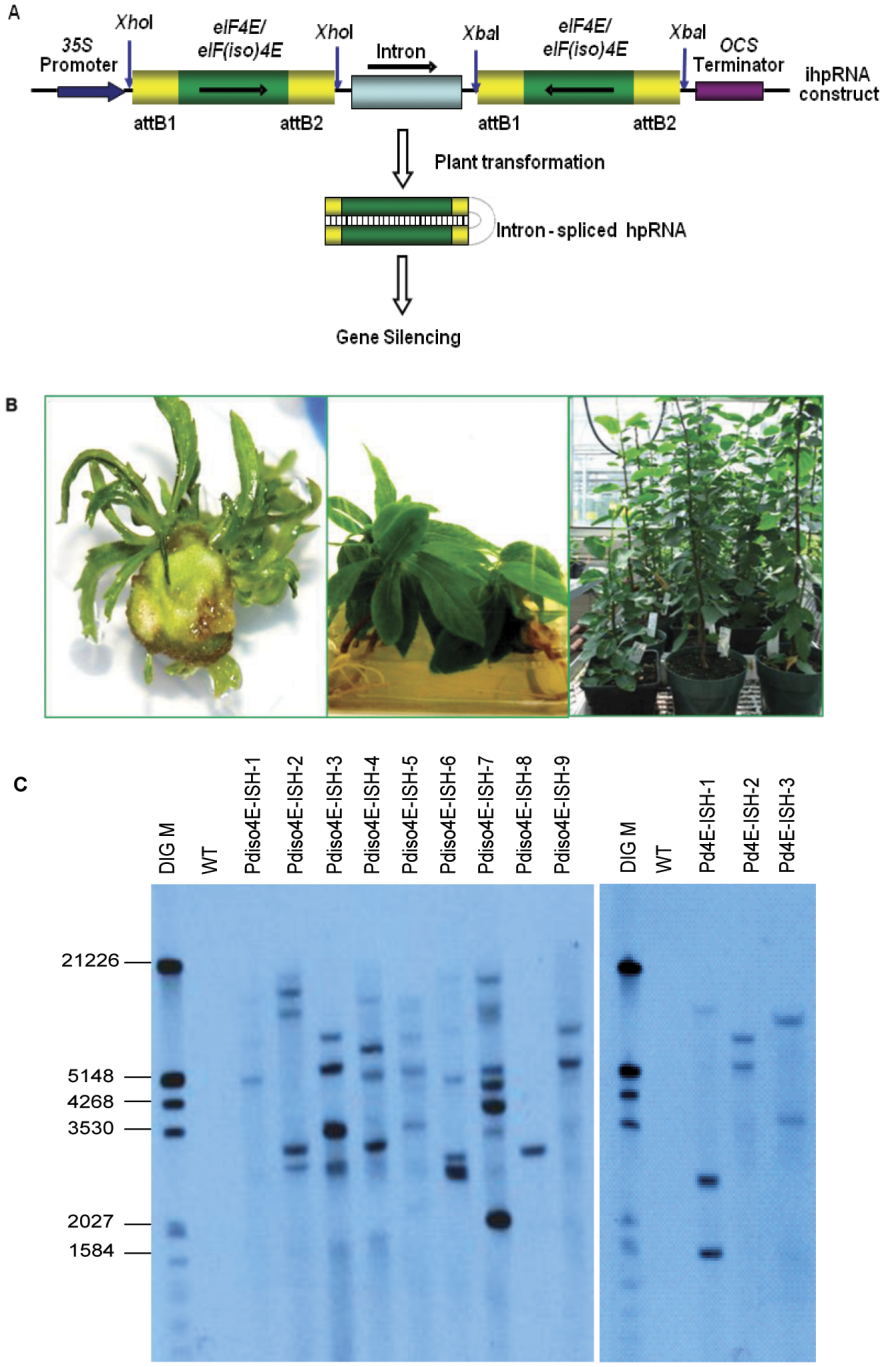
Production and initial analysis of transgenic plum plants. A. Schematic diagrams for *PdeIF4E/eIF(iso)4E* ihp-RNA constructs. Two PCR fragments of *PdeIF4E* or *PdeIF(iso)4E* are present in opposite orientations as indicated by arrows. Transgene expression is under the control of a 35S promoter (large black arrow) and an octopine synthase gene (*OCS*) terminator (purple box). The vertical blue arrows indicate restriction sites used to release the inserts. B. Regeneration of plum transformants. Left: Multiple shoots induced on the shoot induction medium. Middle: Putative transformants rooted in Magenta box. Right: Transgenic plants growing in the greenhouse. C. Southern blot analysis of putative transgenic plums. Genomic DNA was isolated from leaf tissue of transgenic plum plants and digested with *Bam*HI. DNA blot was probed with DIG-labelled *NPT*II probe. DIG M: DIG labelled molecular weight marker (Roche), size shown on the left in bp. WT: Non-transformed wild type plum plant.

To study the involvement of PdeIF4E and PdeIF(iso)4E in PPV infection, we used a gene silencing strategy. DNA constructs containing self-complementary hairpin structure of target genes were developed. It has been previously reported that when both *eIF4E* and *eIF(iso)4E* genes are silenced, plants exhibit a semi-dwarf phenotype [49]. To avoid simultaneous silencing of both genes, sequences which were most divergent between *eIF4E* and *eIF(iso)4E* were selected to design the gene silencing constructs (nts 1–300 for *PdeIF4E* and nts 1–272 for *PdeIF(iso)4E*). Each resulting construct contains the target gene sequences in forward and reverse orientations separated by an intron (Fig. 3A). The constructs were designated pPd4E-ISH and pPdiso4E-ISH.

Putative transgenic shoots formed on the selection medium after four weeks. The developed shoots were excised from explants and cultivated on the shoot regeneration medium supplemented with selective agent for shoot propagation. Induced shoots showed vigorous and continuous growth on the selection medium (Figure 3B). At least five clones of each transgenic line were kept on the selection medium for propagation and the rest of the induced shoots were transferred to rooting medium. After 4–6 weeks, well rooted plantlets were transferred to Magenta boxes and then to the greenhouse for acclimatization (Figure 3B).

Tissue from putative transformants were analysed by PCR using primer 35S-F, located in the CaMV35S promoter region, and gene specific primers Pd4E-siR for *PdeIF4E* and Pdiso-siR for *PdeIF(iso)4E*, respectively. The presence of the Neomycin Phosphotransferase (*NPT*II) gene in transgenic plants was also analysed using primers nptII-F and nptII-R (data not shown).

Southern blot was used to confirm transgene integration into the genome of putative transformants using a probe specific for *NPT*II. DNA samples from all selected transgenic plants showed hybridization with DIG-labelled *NPT*II probe. One to seven hybridization signals were detected in the different plants analysed (Figure 3C), suggesting that most transgenic lines are the result of multiple transgene insertions. Different transgenic plants showed different hybridization patterns indicating that they were the result of independent transformation events and random integration of the transgene. No hybridization signal was detected in non-transformed control plants. In total, 11 *PdeIF(iso)4E* transgenic lines and 6 *PdeIF4E* transgenic lines were obtained. These lines were designated Pdiso4E-ISH and Pd4E-ISH, respectively.

### Small RNA detection and reduced expression of target genes in transgenic plum plants

**Figure 4:**
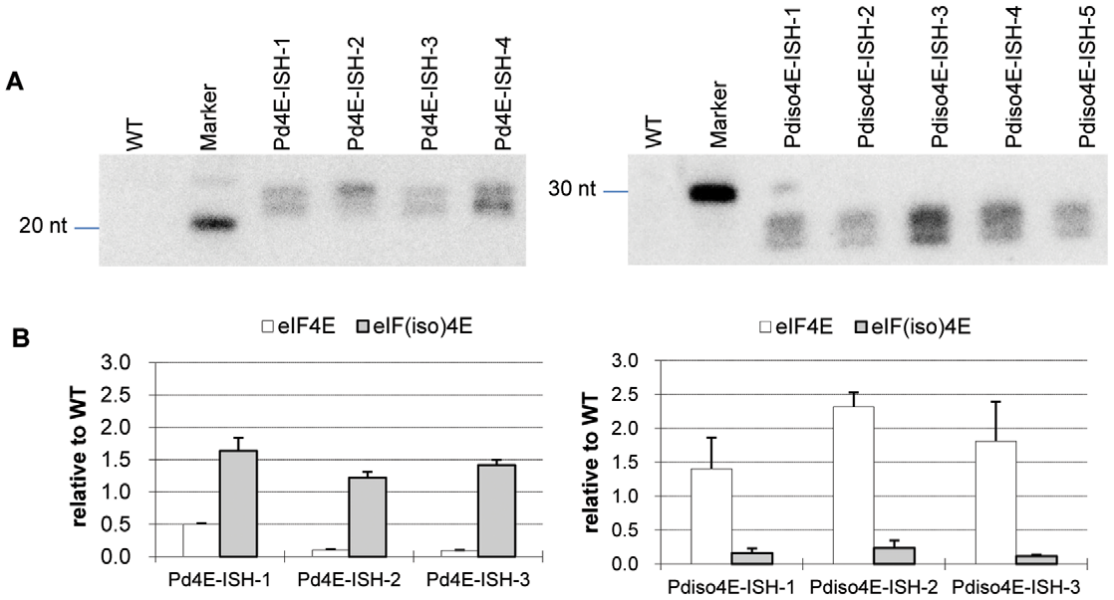
Gene silencing selectively reduces *PdeIF4E* or *PdeIF(iso)4E* transcript levels in transgenic plum plants. A. siRNAs analysis of transgenic plum plants. WT: Non-transformed plum plant. Marker: synthesized short sequences of *PdeIF4E* and *eIF(iso)4E* transgenes, respectively. Left panel: siRNAs in Pd4E-ISH transgenic plants. Right panel: siRNAs in Pdiso4E-ISH transgenic plants. B. *PdeIF4E* and *PdeIF(iso)4E* mRNA expression levels in transgenic plum plants. Transcript levels were analyzed by qRT-PCR. All values were normalized to the reference gene of *Ps-actin* and then compared to the wild type gene expression levels. Error bars represent the standard deviation of two biological replicates each analyzed in triplicate. Left: *PdeIF4E* and *Pdei(iso)4E* transcript levels in different Pd4E-ISH transgenic plants. Right: *PdeIF4E* and *Pdei(iso)4E* transcript levels in different Pdiso4E-ISH transgenic plants.

The presence of *PdeIF4E* or *PdeIF(iso)4E* transgene-specific siRNAs was determined in representative transgenic lines via Northern blot using ^32^P labelled probes specific for each transgene. Previous reports showed that two classes of siRNAs, 21–22 bp and 24–26 bp are usually present in transgenic plants [50]. While both species of siRNAs were detected in all tested transgenic plum plants, no siRNAs were detected in wild type control plants (Figure 4A).

Expression of *PdeIF4E* and *PdeIF(iso)4E* genes in representative transgenic plum lines was investigated by qRT-PCR. In Pd4E-ISH lines, *PdeIF4E* mRNA transcript levels decreased significantly (50%–90% reduction compared to wild-type plants), and in contrast, *PdeIF(iso)4E* levels increased (122% to 164% relative to the wild type plants expression levels) (Figure 4B). Similarly, in Pdiso4E-ISH lines, *PdeIF(iso)4E* gene expression level decreased by 76–88% compared to wild-type plants and the *PdeIF4E* expression level increased to 141% to 230% of the wild-type level (Figure 4B).

The results indicated that the pPd4E-ISH and pPdiso4E-ISH constructs were effectively silencing the expression of the respective target gene. The silencing was specific and silencing of one isoform of *eIF4E* did not result in a decrease of expression of the other isoform. Pd4E-ISH and Pdiso4E-ISH transgenic plants did not exhibit any morphology changes when compared to wild type plants (data not shown).

### PPV resistance

Transgenic plum plants were inoculated with the PPV-D strain by chip-budding [22]. The PPV-infected plants were maintained in a PPV containment room and grown for 4–6 weeks. The plants went through a cold treatment for three months to break the dormancy. The presence of the PPV viral genome in inoculated plum plants was tested by direct real time PCR [51]. All tests were conducted by performing three technical replicates for each plant. Samples with a threshold cycle (Ct) higher than 36 or undetectable was considered to be PPV negative as outlined in our previous research [51]. After the first cycle of cold treatment, 26 out of 28 Pdiso4E-ISH plants were virus free. In contrast, 15 out of 16 Pd4E-ISH plants and all 5 non-transformed control plants were PPV positive (Table 2).

**Table 2:** PPV resistance based on drt-PCR analysis of the absence of PPV genome.

			PPV Negative			
	Transgenic lines	No. of plant tested	Cold treatment 1		Cold treatment 2	
			No. of plant	%	No. of plant	%
*eIF(iso)4E* transgenic lines	Pdiso4E-ISH-1	4	4	100	4	100
	Pdiso4E-ISH-2	5	5	100	4	80
	Pdiso4E-ISH-3	3	3	100	3	100
	Pdiso4E-ISH-4	1	1	100	1	100
	Pdiso4E-ISH-5	2	2	100	2	100
	Pdiso4E-ISH-6	3	3	100	2	66.7
	Pdiso4E-ISH-7	1	1	100	0	0
	Pdiso4E-ISH-8	2	2	100	2	100
	Pdiso4E-ISH-9	1	1	100	1	100
	Pdiso4E-ISH-10	2	1	50	1	50
	Pdiso4E-ISH-11	4	3	75	3	75
Total		28	26	6	23	82
*eIF4E* transgenic lines	Pd4E-ISH-1	5	0	0	0	0
	Pd4E-ISH-2	3	0	0	0	0
	Pd4E-ISH-3	3	0	0	0	0
	Pd4E-ISH-4	1	0	0	0	0
	Pd4E-ISH-5	1	0	0	0	0
	Pd4E-ISH-6	3	1	33	*	0**
Total		16	1	6	0	0
Wild type		5	0	0	0	0

%: percentage resistance after each of two cycles of cold treatment.

* plant died

** percentage of resistance excluding dead plants.

To test the stability of PPV resistance, the plants went through a second cycle of cold treatment in which the plants were placed in the cold room for three months. After the cold treatment, the plants were brought back to the greenhouse with normal growth conditions. The presence of PPV was tested 6 and 16 weeks after the second cold treatment. All Pd4E-ISH plants that tested positive in the first test remained PPV positive. All control plants tested PPV positive and exhibited stunted growth. As for Pdiso4E-ISH lines, only 3 of the 26 plants that tested PPV negative after the first cold treatment became PPV positive after the second cold treatment (Table 2). In total, 82% of Pdiso4E-ISH plants remained virus free after two cycles of cold treatment (Table 2).

**Figure 5:**
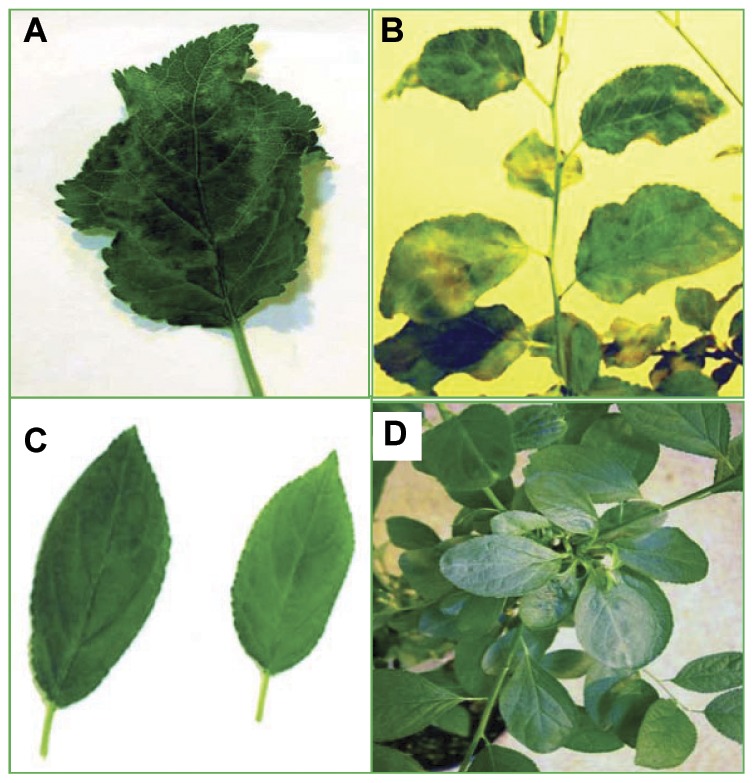
PPV symptoms on infected plum plants and absence of symptoms on resistant Pdiso4E-ISH transgenic plants. A. Ring spots on susceptible transgenic plum leaf. B. Chlorotic spots on wild type plum tree leaves. C. PPV resistant Pdiso4E-ISH transgenic plum leaves. D. PPV resistant Pdiso4E-ISH transgenic plants.

PPV symptoms were visually monitored after cold treatments. Only two wild type plants exhibited typical PPV symptoms, e.g. chlorotic spots and ring patterns, on leaves (Figure 5), four weeks after the first cycle of cold treatment. All wild type plants and 12 Pd4E-ISH plants exhibited PPV symptoms six weeks after the second cold treatment. PPV symptoms were also seen on the few Pdiso4E-ISH plants that tested PPV positive. PPV-resistant Pdiso4E-ISH plants remained asymptomatic.

### Interaction between PdeIF(iso)4E and PPV-VPg

**Figure 6:**
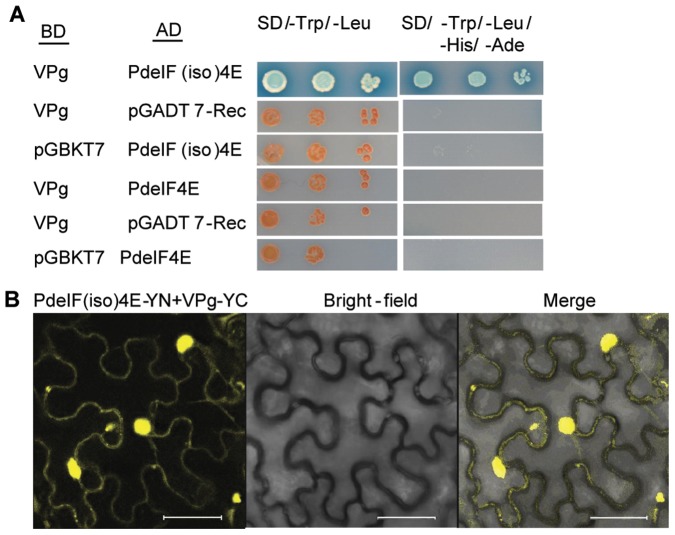
Protein interaction between PdeIF(iso)4E and PPV-VPg. A. Y2H assay showing the interaction between plum eIF(iso)4E and PPV-VPg. BD and AD represent genes fused with GAL4 binding and activation domains, respectively. pGBKT7 and pGADT7-Rec represent empty vectors for bait and prey, respectively. SD/-Trp/-Leu and SD/-Trp/-Leu/-His/-Ade correspond to double dropout medium lacking Trp and Leu and quadruple dropout medium lacking Trp, Leu, His and Ade, respectively. Positive interactions result in yeast growth on the SD/-Trp/-Leu/-His/-Ade plus X-α-Gal plate and the development of the blue color. B. BiFC images confirming the positive interactions shown by Y2H. -YN, protein fused to N-terminal YFP; -YC, protein fused to C-terminal YFP. Scale bars = 38 µm.

To determine if there is an interaction between PPV-VPg and eIF4E/eIF(iso)4E, the GAL4 based yeast two hybrid (Y2H) system was used. Reporter gene activation (*ADE*, *HIS*, *MEL1*) was only observed when VPg and eIF(iso)4E were cloned into the bait and prey vectors and expressed in the same cell. This was evidenced by the ability of these cells to grow in absence of adenine and histidine and the development of the blue color (Figure 6A). In contrast, activation of reporter genes was not observed when VPg was co-expressed with eIF4E. As expected, expression of either VPg, eIF(iso)4E or eIF4E GAL fusion proteins with the corresponding unfused GAL4-BD or GAL4-AD protein (negative controls) did not result in the activation of any of the reporter genes.

To test if the positive interaction between PPV-VPg and PdeIF(iso)4E can be observed *in planta*, protein interactions were analyzed using the bimolecular fluorescence complementation (BiFC) assay. The PPV-VPg protein was fused to the C-terminal fragment of the yellow fluorescent protein (YFP) and the PdeIF(iso)4E protein was fused to the N-terminal fragment of YFP. Following transient co-expression of PPV-VPg-YC and PdeIF(iso)4E-YN in *Nicotiana benthamiana*, interaction of these proteins allowed reconstitution of fluorescence (Figure 6B). This interaction was observed throughout the cytoplasm (Figure 6B) and nucleus (not shown). No interaction was observed between VPg and eIF4E. Using both Y2H and BiFC assays, the results indicated an interaction between PPV-VPg and PdeIF(iso)4E but not between PPV-VPg and PdeIF4E. This was consistent with the observation that silencing of *PdeIF(iso)4E* in plum can confer resistance to PPV, while silencing of *PdeIF4E* did not. Thus, the VPg-eIF(iso)4E interaction is probably important for PPV infection in plum.

## Discussion

In this study, we have shown that PPV resistance can be efficiently achieved in a PPV natural host plant by silencing *eIF(iso)4E* expression using an RNA silencing-based strategy. The resistance was stable over an extended period of time and over two growth and dormancy cycles.

Both eIF4E and eIF(iso)4E are part of the eIF4F complex and function in recruiting mRNAs to ribosomes for translation initiation [52]–[54]. It has been proposed that the two isoforms of eIF4F have different biological functions in regulating plant growth and development [55]. eIF4F and eIF(iso)4F have been shown to discriminately recruit different types of mRNAs for translation during plant growth and development [37]. While eIF4F supports translation of mRNAs containing 5′-proximal secondary structure and uncapped mRNAs better than eIF(iso)4F, eIF(iso)4F preferentially recruits unstructured mRNAs [37], [38], [54]. This suggests that the two isoforms may have distinct functions in cell development and metabolism. We investigated the mRNA transcription profiles of *eIF4E* and *eIF(iso)4E* in PPV nature woody host plant (plum). We did not find significant difference in the expression profiles of the two genes in different tissues. Both genes were transcribed abundantly in leaves, leaf buds and flower buds, and were expressed at a relatively lower level in petals (Figure 2). This suggests that both genes are needed for plant growth and development under normal conditions. This result is consistent with previous research conducted in *Arabidopsis*, which showed that both genes are highly expressed in meristematic tissues and in actively dividing cells in immature organs, but were differentially expressed in other types of mature tissue [56].

Mutant plants lacking either eIF4E or eIF(iso)4E do not show phenotype change compared to wild type plants, suggesting some degree of functional redundancy between the two isoforms [29], [33], [43], [49], [57]. This functional redundancy, however, does not extend to virus infection, since viruses appear to selectively recruit one member of the eIF4E family for their infection. Many recessive viral resistance genes (primarily for potyviruses) encode isoforms of the subunits of eIF4F and eIF(iso)4F, especially eIF4E and eIF(iso)4E [35], [39]. eIF4G and eIF(iso)4G have also been linked to virus resistance [34], [58]. The specificity for either eIF4E or eIF(iso)4E appears to depend on the specific virus and host considered. In *Arabidopsis*, an eIF4E mutant line confers resistance to *Clover yellow vein virus* (ClYVV), while eIF(iso)4E mutant lines are resistant to *Turnip mosaic virus* (TuMV) and *Tobacco etch virus* (TEV) [33]. Moreover, the same virus may recruit different isoforms of eIF4E to replicate in different plant species. For example, TEV depends on eIF4E for infection in pepper, tomato and lettuce [30], [32], [42], but depends on eIF(iso)4E in *Arabidopsis* [29], [31]. Some viruses can use both isoforms for multiplication as shown in pepper for *Pepper veinal mottle virus* (PVMV) [59]. The specificity can also vary with virus isolates [60]. All PPV strains tested failed to infect an eIF(iso)4E knockout mutant in *Arabidopsis* [46]. This is a strong indication that eIF(iso)4E plays an essential role in PPV infection in this host. However, before this study was initiated, it was not certain that eIF(iso)4E was also playing a role in PPV infection in natural PPV host woody plant. In the present study, the involvement of both eIF4E and eIF(iso)4E in PPV infection was investigated by testing PPV resistance in transgenic plum plants lacking either eIF4E or eIF(iso)4E. Eighty-two percent of transgenic plum plants expressing hairpin RNA targeting *eIF(iso)4E* were resistant to PPV-D over an extended period of time involving two growth and dormancy cycles (Table 2). In contrast, all Pd4E-ISH transgenic plants were susceptible to PPV. VPg of potyviruses serves as a primer for viral RNA synthesis. The interaction between VPg and eIF4E/eIF(iso)4E has been shown to be important for potyvirus infection and amplification [30], [41]. However, the details and the mechanism of the interaction in terms of viral infection are not clear yet. Depending on the virus and on the plant species, VPg either interacts with eIF4E or eIF(iso)4E and the disruption of the interaction leads to the virus resistance [61]–[63]. The Y2H and BiFC analysis results in this study showed there is a physical interaction only between PdeIF(iso)4E and PPV-VPg (Figure 6). The results also reveal that silencing of *eIF(iso)4E* gene is an effective approach for generating host factor based resistance to PPV.

Disruptions of gene expression can be achieved by the use of a T-DNA insertions or transposon deletion mutants. There are limitations in using these techniques, including the lack of an appropriate transposon system or the difficulties associated with establishing T-DNA lines in some plant species. Both concerns are especially true in perennial woody tree plants. Moreover, identification of insertion lines for a specific gene can be time consuming and labour extensive. RNA silencing functions in a sequence-specific manner, and therefore, can specifically silence a target gene. Plant lines where individual genes have been silenced can be quickly generated using transgenic technology. The specificity allows the silencing of only one sequence among closely related sequences, as seen in this study, where only one isoform of *eIF4E* gene was effectively silenced (Figure 4B). Similar specific silencing of one isoform of eIF4E was also recently described in melon and tomato [64], [65]. The use of intron-containing self-complementary hpRNA (ihp-RNA) constructs was reported to dramatically enhance the RNA silencing efficiency up to 100% [66], [67]. Use of ihp-RNAs to silence viral gene to achieve PPV resistance has been documented in herbaceous species and *Prunus* species [17]–[21] using pathogen-derived resistance approaches. In this study, PPV resistance was obtained by expressing ihp-RNAs targeting an essential host factor (eIF(iso)4E) in plum. Such resistance eliminates potential concerns about the presence of the viral sequence in transgenic plants and provide an attractive alternative strategy to engineer virus resistance.

In this study, we have shown that eIF(iso)4E is involved in PPV infection in its natural host woody plant and that silencing of *eIF(iso)4E* gene expression can effectively confer PPV resistance to a *Prunus* plant. To our knowledge, this is the first demonstration that manipulation of the expression of a host factor can provide an effective resistance source for the control of PPV in woody trees. It is likely that the transgenic plants are also resistant to other strains of PPV or to other viruses recruiting the same host factor for infection. Additional tests will answer this question.

## Experimental Procedures

### Cloning and sequencing of plum *eIF4E* and *eIF(iso)4E* genes

Tissues from plum (*Prunus domestica* L.) were collected from the orchard of the University of Guelph, Vineland Station, Ontario, Canada. Unless stated otherwise, young leaves were collected and used for RNA and DNA extractions. Four to five leaf discs (approximately 50∼100 mg) from each plant were collected and immediately frozen in liquid nitrogen. Leaf tissues were homogenized with a Mixer Mill MM 300 (Retsch, Haan, Germany). A Plant RNA Isolation Kit (Norgen Biotek Corp., Thorold, ON, Canada) was used for RNA extraction. The genomic DNA was removed using DNase I (New England Biolabs, Inc., Ipwich, MA, USA) before RNA was eluted according to the manufacturer instructions. siRNAs were isolated from young leaves using the MicroRNA Purification Kit (Norgen Biotek Corp.). One microgram of purified total RNA was used for cDNA synthesis using the QuantiTect® Reverse Transcription kit (Qiagen GmbH, Hilden, Germany). The RT reactions were incubated at 42°C for 30 min, followed by 3 min at 95°C to inactivate the reverse transcriptase. This was followed by standard PCR.

To obtain the 5′- and 3′- sequences of plum *eIF4E* and *eIF(iso)4E* genes, 5′-RACE and 3′-RACE were carried out using the First Choice RLM-RACE kit (Ambion Inc., Austin, TX, USA) following the manufacturer's instructions. The *eIF4E* gene specific 5′-RACE and 3′-RACE primers, Pd4E-5′-RACE-OP, Pd4E-5′-RACE-IP, Pd4E-3′-RACE-OP and Pd4E-3′-RACE-IP (Table 1), were used for amplifying plum *eIF4E*. Similarly, the *eIF(iso)4E* gene-specific 5′-RACE and 3′-RACE primers, Pdiso4E-5′-RACE-OP, Pdiso4E-5′-RACE-IP, Pdiso4E-3′-RACE-OP and Pdiso4E-3′-RACE-IP (Table 1), were used for plum *eIF(iso)4E* amplification. Purified PCR fragments were cloned into pGEM T-easy vector (Promega, Madison, WI, USA) and sequenced.

### Analysis of plant gene expression by quantitative real-time PCR (qRT-PCR)

Total RNA samples were extracted from root, stem, leaf, petal, immature green fruit, flower bud, anther and leaf bud tissues, and reverse transcribed to cDNAs. Tissues used for RNA extraction were pooled from three plum plants. For mRNA transcript level analysis, three RNA samples were isolated for each type of tissues and used as template for biological repeats. For transgenic plants, three RNA samples were isolated for each plant using leaf tissues pooled from the same plant.

qRT-PCR reaction preparations were conducted by using the QuantiTect® SYBR® Green PCR Kit (Qiagen GmbH, Hilden, Germany) on a LightCycler® 480 real-time PCR system (Roche Diagnostics GmbH, Mannheim, Germany). Primer sets Pd4E-Fm3 and Pd4E-Rm3, and Pdiso-Fm3 and Pdiso-Rm3 were used for *PdeIF4E* and *PdeIF(iso)4E* amplification, respectively. The *ACTIN* gene from *Prunus salicina* L. was used as the internal reference gene using primers Ps-actin-F and Ps-actin-R [48] (Table 1). For each primer pair, gel electrophoresis was performed to ensure that a single PCR product of the expected size was generated. A melting curve analysis was also conducted for each primer pair. Each reaction contained 12.5 ng of cDNA template, 0.3 mM primer mix and 5 µl of CYBR Green Master mix in a total volume of 10 µl. Reactions were carried out as follows: pre-incubation at 95°C for 10 min, followed by 40 cycles of 94°C for 10 sec and 56°C for 30 sec, and a single cycle as an extension at 72°C for 30 sec before performing a melting curve analysis. For each sample analyzed, there were three biological replicates, and for each replicate, there were three technical repeats. Samples were run on a 96-well-plate (Roche). All qRT-PCR data were acquired and processed using the LightCycler® 480 Software1.5.0 SP3 (Roche). Relative expression values were obtained by interpolating experimental reactions in standard curves which were created using a cDNA template for *PdeIF4E*, *PdeIF(iso)4E* or plum *ACTIN* genes. Each standard curve composed of 15 datapoints representing 3 replicates for 5 different DNA concentrations of 2× dilution series starting from 12.5 ng. The reaction efficiency for all standard curves was greater than 95%. The level of each mRNA was calculated using the mean Ct normalized to the corresponding reference gene for gene expression profiles in different tissues. All results are shown as means of the three biological replicates with corresponding standard errors.

### Construction of intron-spliced-hairpin constructs

Plasmid constructs containing ihp-RNAs of target genes were cloned using the bacteriophage lambda site-specific recombination based Gateway® technology [68]. First, genes of interest were amplified by PCR using gene specific primers with flanking *att*B1 and *att*B2 sites, Pd4E-attB1 and Pd4E-attB2 for *Pd4E* and Pdiso-attB-1 and Pdiso-attB-2 for *PdeIF(iso)4E* (Table 1). Purified PCR fragments were cloned into the pDONR221 vector through BP reaction using BP® clonase (Invitrogen, Carlsbad, CA, USA) to make an entry clone. Entry clones were confirmed by sequencing and integrated into the destination vector pHELLSGATE12 [67] by LR recombination reaction using the LR clonase™ enzyme mix (Invitrogen) to make the expression clone. Constructed plasmids were transformed into *Agrobacterium tumefaciens* strain EHA105 [69] by electroporation.

### Plum transformation

Plum hypocotyls of mature seeds were used as explants for transformation and the procedure was as described previously [70]. When more than one shoot developed from an explant, only one shoot was used for further analysis.

### Screening of transgenic plants by PCR and Southern blot analysis

DNAs isolated from putative transgenic plants were used as templates and the PCR was conducted with Taq DNA polymerase (Genscript) reaction with primers CTAP and Pd4E-siR and CTAP and Pdiso-siR respectively in 20 µl PCR reaction. CTAP primer is located at the 35S promoter and the reverse primers Pd4E-siR and Pdiso-siR are sequence specific. Southern blots were conducted using DIG High Prime DNA Labelling and Detection Starter Kit II (Roche Diagnostics GmbH, Mannheim, Germany) as described by Tian *et al.*, (2009), except that genomic DNA samples were extracted from transgenic plum plants using the Plant DNA Isolation kit (Norgen Biotek, Thorold, ON. Canada) and each DNA sample was digested with *Bam*HI (New England Biolabs, Inc., Ipwich, MA, USA).

### Detection of small interfering RNAs (siRNAs)

Detection of small interfering RNAs was performed as described (Zhang *et al.*, 2006) with the following modifications. Approximately 8 µg of small RNA samples were mixed with 2× RNA loading buffer (Norgen Biotek, Thorold, ON, Canada) and separated on a 7 M urea denatured 20% polyacrylamide gel in 0.5× TBE buffer ran for approximately 90 min at 180 V in a BIORAD Mini-Protein Cell (Bio-Rad, Hercules, CA, USA). The RNAs were transferred to Hybond-N^+^ nylon membrane (GE Healthcare Limited, Buckinghamshire, UK) using semi-dry transfer cell (Bio-Rad, Hercules, CA, USA) at 180 mA for approximately 2 h. The RNAs were fixed to the membrane by UV cross-linking. DNA fragments corresponding to the entire length of the transgenes were amplified from plasmids containing *PdeIF4E* and *PdeIF(iso)4E* coding sequences, respectively, by PCR using primers Pd4E-F8 and Pd4E-siR, and Pdiso-F1 and Pdiso-siR (Table 1). The amplified fragments were radiolabelled as probes and were hybridized to the RNA blots as described by Zhang *et al.* (2006).

### Yeast two hybrid (Y2H) assay

Protein interactions were tested using the Matchmaker Gold Yeast Two-Hybrid System (Clontech, Mountain View, CA, USA). *PdeIF4E* and *PdeIF(iso)4E* coding sequences were amplified using primers Pd4E-attB1/Pd4E-attB2-Y2H and Pdiso-attB1/Pdiso-attB2-Y2H (Table 1) and the VPg coding sequence was amplified from PPV-YN plasmid [71] using primers VPg-attB1/VPg-attB2 (Table 1). All sequences were amplified by PCR using Phusion High fidelity DNA polymerase (New England Biolabs, Inc., Ipwich, MA, USA) and cloned into two Gateway-compatible Y2H vectors pGBKT7-DEST (bait) and pGADT7-DEST (prey) [72]. The *PPV-VPg* and *PdeIF4E* and *PdeIF(iso)4E* cDNA sequences were cloned into both bait and prey vectors, but only results using PPV-VPg as a bait and PdeIF4E or PdeIF(iso)4E as prey are shown. Reciprocal assay using PPV-VPg as a prey and PdeIF4E or PdeIF(iso)4E as bait gave the same results. For co-transformation of bait and prey constructs, yeast strain Y2HGold was used according the Yeastmaker Yeast Transformation System protocol (PT1172-1, Clontech, Mountain View, CA, USA). After transformation, cells were spread on SD double drop out (DDO), SD/-Trp/-Leu, agar plates and incubated at 28°C for 3–5 days. Colonies growing on DDO plates were suspended in DDO liquid medium. A 10× dilution series of 5 µl aliquots of co-transformed Y2HGold were spotted onto DDO/X (DDO medium supplemented with X-α-Gal to test for the expression of the *MEL1* marker) and QDO/X/A (QDO medium supplemented with X-α-Gal and aureobasidin A) agar plates. Plates were incubated at 28°C for 3–5 days. Colonies growing on the DDO plates were analyzed following the manufacturer's protocol (Clontech).

### BiFC assay

The Gateway compatible BiFC vectors pEarleygate201-YN and pEarleygate202-YC [72], containing amino acids 1–174 and 175–239 of YFP, respectively, were used. The *PdeIF4E* and *PdeIF(iso)4E* cDNA sequences were amplified using the primer pairs listed Table 1 and fused in frame with the coding region of the N-terminal fragment of YFP generating constructs eIF4E-YN and eIF(iso)4E-YN. *PPV-VPg* cDNA sequences were cloned to be expressed as fusion to the C-terminal fragment of YFP (construct VPg-YC). All YFP fusion constructs were transformed into *A. tumefaciens* strain GV3101. To infiltrate epidermal *N. benthamiana* cells, *Agrobacterium* cultures were grown to an OD_600_ of 1.0. Equal volumes of each culture were mixed and infiltrated in *N. benthamiana* leaves as described [73]. The YFP signal was imaged 36–48 hours after infiltration using a Leica TCS SP2 confocal microscope (Leica Microsystems Heidelberg GmbH, Mannheim, Germany).

### PPV resistance evaluation

Plum plants were inoculated with PPV strain D virus by chip-budding [22]. Transgenic plum plants and five non-transgenic regenerated plum plants were inoculated by inserting three bud chips from PPV-D strain infected peach trees onto the plum stems. PPV-infected plants were maintained in a PPV containment room and grown at 21–23°C with 16 h light period for 4–6 weeks. This was followed by a dormant period at 4°C in the dark for three months. Following dormancy, plants were moved to the growth room with regular temperature and light length. The stability of the resistance phenotype was examined by going through a second dormancy/growth cycle. Four to six weeks after the cold treatment, PPV-infected trees were tested by direct real-time RT-PCR to check for the presence of the virus as described [51].

### *In silico* analysis

eIF4E and eIF(iso)4E protein sequences were identified by BLASTP searches at NCBI (http://blast.ncbi.nlm.nih.gov). cDNA sequences assembling, genomic DNA alignments and translation of nucleotide to protein sequences were performed with SeqMan, MegaLign and EditSeq programs of Lasergene 6. Full length amino acid sequences from select plant species andhuman were aligned (DNAman Version 6) using an optimal sequence alignment and a PAM protein weight matrix [74]. The phylogenetic tree was generated using a Maximum-Likelihood method based on the Jones-Taylor-Thornton model (DNAman Version 6).
